# TMS of the left primary motor cortex improves tremor intensity and postural control in primary orthostatic tremor

**DOI:** 10.1007/s00415-024-12376-3

**Published:** 2024-04-16

**Authors:** Florian Schoeberl, James Dowsett, Cauchy Pradhan, Denis Grabova, Angelina Köhler, Paul Taylor, Andreas Zwergal

**Affiliations:** 1 Department of Neurology and German Center for Vertigo and Balance Disorders (DSGZ), LMU University Hospital, LMU Munich, Marchioninistrasse 15, 81377 Munich, Germany; 2 German Center for Vertigo and Balance Disorders (DSGZ), LMU University Hospital, LMU Munich, Munich, Germany; 3 Division of Psychology, University of Stirling, Stirling, UK; 4 Faculty of Philosophy, Philosophy of Science and the Study of Religion, LMU Munich, Munich, Germany

**Keywords:** Primary orthostatic tremor, Transcranial magnetic stimulation, Primary motor cortex, Imbalance, Dizziness, Posturography, Sway path

## Abstract

A ponto-cerebello-thalamo-cortical network is the pathophysiological correlate of primary orthostatic tremor. Affected patients often do not respond satisfactorily to pharmacological treatment. Consequently, the objective of the current study was to examine the effects of a non-invasive neuromodulation by theta burst repetitive transcranial magnetic stimulation (rTMS) of the left primary motor cortex (M1) and dorsal medial frontal cortex (dMFC) on tremor frequency, intensity, sway path and subjective postural stability in primary orthostatic tremor. In a cross-over design, eight patients (mean age 70.2 ± 5.4 years, 4 female) with a primary orthostatic tremor received either rTMS of the left M1 leg area or the dMFC at the first study session, followed by the other condition (dMFC or M1 respectively) at the second study session 30 days later. Tremor frequency and intensity were quantified by surface electromyography of lower leg muscles and total sway path by posturography (foam rubber with eyes open) before and after each rTMS session. Patients subjectively rated postural stability on the posturography platform following each rTMS treatment. We found that tremor frequency did not change significantly with M1- or dMFC-stimulation. However, tremor intensity was lower after M1- but not dMFC-stimulation (*p* = *0.033*/* p* = *0.339*). The sway path decreased markedly after M1-stimulation (*p* = *0.0005*) and dMFC-stimulation (*p* = *0.023*) compared to baseline. Accordingly, patients indicated a better subjective feeling of postural stability both with M1-rTMS (*p* = *0.007*) and dMFC-rTMS (*p* = *0.01*). In conclusion, non-invasive neuromodulation particularly of the M1 area can improve postural control and tremor intensity in primary orthostatic tremor by interference with the tremor network.

**Supplementary Information:**

The online version contains supplementary material available at 10.1007/s00415-024-12376-3.

## Introduction

Orthostatic tremor (OT) is a rare and frequently unrecognized movement disorder first described in 1970 by Pazzaglia and eventually denoted as a distinct tremor syndrome in 1984 by Heilman [1, 2]. OT is characterized by a synchronous tremor of homologous muscles on both legs with a high frequency of 13–18 Hz during standing [3, 4]. The diagnostic gold standard is surface electromyography (EMG) on homologous muscle pairs of both legs, e.g. tibialis anterior and gastrocnemius [3, 5, 6]. Diagnosis is delayed up to several years in most patients due to general physicians not being aware of the typical complaints, i.e., patients being unsteady standing and better walking [7–10]. OT can be differentiated into primary OT (POT) [8, 9], without underlying aetiology and in the absence of structural brain lesions, or secondary OT, which was described in patients with pontine brainstem or cerebellar lesions or in neurodegenerative disorders with cerebellar atrophy [10–12]. POT is a progressive condition with worsening of symptoms and increase in body sway, with effects extending to the trunk and arms over time [8, 9, 13]. In at least half of the cases with POT, treatment with different drugs such as clonazpeam, gabapentine, and ß-blockers has no satisfactory effect [6, 8]. Deep brain stimulation (DBS) of the ventral intermediate nucleus (VIM) of the thalamus or zona incerta (ZI) can lead to a modest reduction of symptoms in pharmacorefractory cases of POT, which diminishes over time [14–17]. However, data are still limited as compared to other tremor disorders.

Recent functional imaging and EEG/EMG coherence studies revealed consistently that a ponto-cerebello-thalamo-cortical tremor network is the pathophysiological correlate of POT [18–20]. Cortical activations in POT are mainly restricted to the paramedian portions of the primary motor cortex (M1), where the legs are represented [18, 20].

The purpose of the present study was to evaluate the effects of repetitive transcranial magnetic stimulation (rTMS) in POT as an additional non-invasive treatment option. We hypothesized that theta burst rTMS of the M1 leg area could selectively downregulate the entire tremor network in POT, which would consequently lead to a decrease of tremor intensity and sway path. Therefore, theta burst rTMS was applied to eight patients with proven POT at two different stimulation sites, i.e., M1 leg area and dorsal medial frontal cortex (dMFC) in a cross-over design. Effects on tremor frequency and intensity were measured by surface EMG on homologuous muscles on both lower legs, and analysis of the frequency spectrum and sway path by posturography, before and after rTMS for both stimulation sites.

## Materials and methods

### Subjects

Eight patients (four females, mean age 70.2 ± 5.4 years) with a definite diagnosis of POT according to the currently accepted diagnostic criteria [3] were included in the study. Past medical and drug history was documented (for details see Table 1). Patients reported a gradual onset of unsteadiness during upright stance, which increased while standing still and disappeared during walking or sitting down. Subjects underwent a standardized neurological examination to exclude additional clinical signs indicative of secondary orthostatic tremor (i.e., hypokinesia, rigidity, dystonia, failure of gait initiation, cerebellar ataxia). Brain MRI was performed in each patient to definitely exclude structural lesions and/or atrophy, particularly in the brainstem and cerebellum. All subjects completed the Beck Depression Inventory II (BDI–II) and the Dizziness Handicap Inventory (DHI) (Table 1).

**Table 1:** Demographic and clinical characteristics of individual patients with POT

ID	Sex	Age	POT duration (years)	Current medication against POT	BDI–II-score	DHI-score
1	F	57	8	Gabapentin 1800 mg/d	19	45
2	F	71	23	Gabapentin 1200 mg/d	24	73
3	M	77	14	Clonazepam 3 mg/d	29	75
4	F	58	11	Gabapentin 900 mg/d	26	58
5	F	68	18	None	11	64
6	M	76	13	Gabapentin 900 mg/d	13	68
7	M	78	15	Propranolol 120 mg/d	17	58
8	M	59	6	Gabapentin 900 mg/d	7	35

*POT* primary orthostatic tremor, *BDI–II* Beck depression inventory II, *DHI* dizziness handicap inventory

### Surface electromyography

Surface EMG recordings were made with Zebris DAB-Bluetooth (Zebris Medical) using bipolar Ag/AgCl electrodes (Noraxon Inc.). The band width of the sampling frequency was 7–500 Hz. The sampling rate was 250 Hz for each EMG electrode. Although the sampling rate was within the pass band, the practical consequences are likely to be limited as the power content of EMG over 125 Hz is low. The obtained data were analysed with MATLAB software (MathWorks Inc., Natik, MA). In all patients with POT, surface EMG was recorded from the anterior tibial and medial gastrocnemius muscles of both legs during lying and upright stance to definitely diagnose POT, exclude additional tremor forms and concurrent differential diagnoses.

### Posturography

Posturographic measurements with increasing difficulty were made in subjects while standing on a Kistler platform. Conditions included standing on firm ground with eyes open (EO), eyes closed (EC), and eyes open on foam rubber (EOF) [21]. EO and EC were used as training sessions, while EOF was considered the test condition for the data analysis. Each session lasted for 30 s. The total sway path was analysed for x and y directions. Fourier analysis was applied to quantify the distribution of frequencies in the frequency spectrum of body sway.

### Theta burst repetitive transcranial magnetic stimulation (rTMS)

Transcranial magnetic stimulation (TMS) was provided using a MagPro R30 machine with a MC-B70 Butterfly Coil (Medtronic). Biphasic pulses were applied either over the left primary motor cortex (M1) or over the dorsal medial frontal cortex (dMFC). M1 was determined individually as the site where TMS elicited a selective twitch in the contralateral lower leg. dMFC was defined as the location on the midline 5 cm anterior to the vertex, which is the midpoint between nasion and inion. In addition, we localised the hand area of M1 as the area providing the most selective contralateral finger twitch. The resting motor threshold (RMT) was taken from the M1 hand area, defined as the minimum TMS intensity required to achieve a visible contraction of the contralateral hand in 5 out of 10 consecutive pulses [22]. The rTMS intensity for the stimulation protocol was set at 80% of the RMT from the hand M1 representation. The foot area is deeper within primary motor cortex than the hand area making the motor threshold for the foot far higher. Safety guidelines for theta burst TMS however tend to assume the hand area is stimulated: to unambiguously satisfy these guidelines we concordantly took the conservative option of using the hand area motor threshold for stimulating both areas in the main experiment (i.e., the M1 representation of the lower limb and the dMFC). The rTMS protocol consisted of 40 s of continuous theta burst stimulation (50 Hz triplets in bursts applied at 5 Hz) [23].

### Procedures

**Fig. 1:**
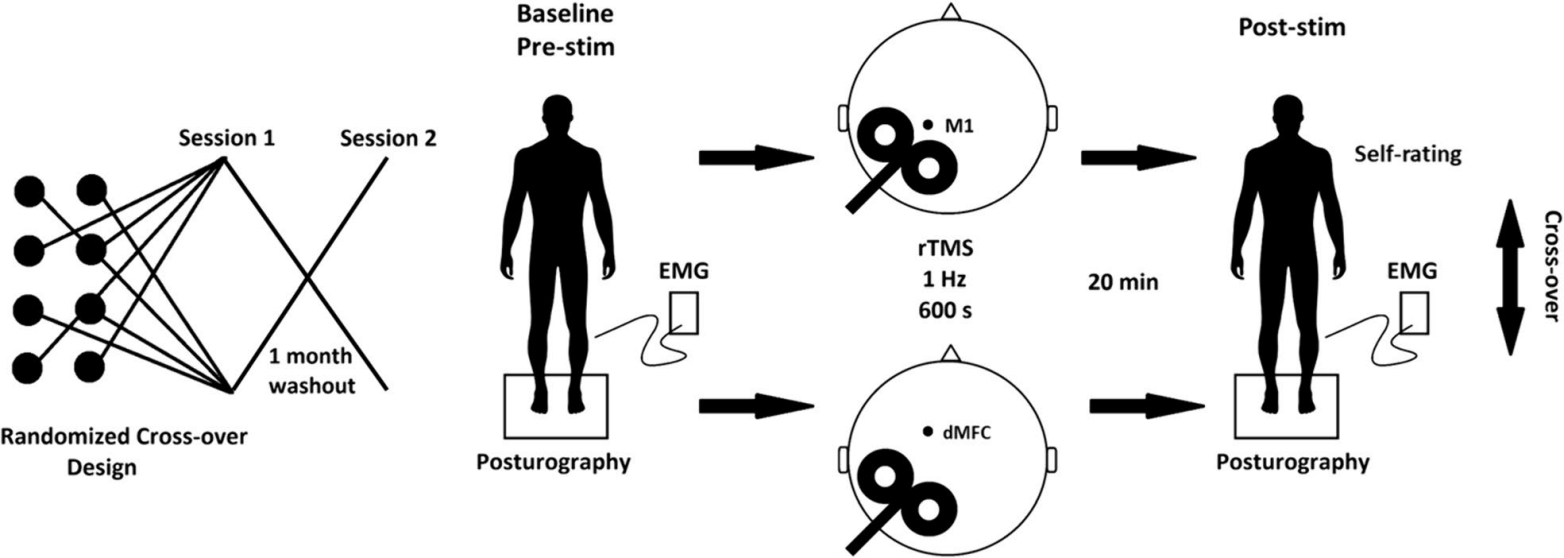
Experimental setup and study design. Each of the eight POT patients underwent theta burst rTMS (1 Hz, 600 s) of the leg area of the left primary motor cortex (M1) and dorsal medial frontal cortex (dMFC). The stimulus intensity was 80% of the M1 hand area motor threshold in each patient. Before stimulation, i.e. at baseline (pre-stim), and 20 min after stimulation (post-stim) tremor frequency (Hz) and intensity (uv.s) was recorded by surface EMG of both Mm. tibialis anterior and gastrocnemius. In addition, the cumulative sway path on a foam rubber when eyes open (EOF) was recorded in each patient pre-stim and post-stim on a Kistler posturography platform for 30 s, respectively. Change in subjectively perceived postural stability was scored by patients post-stim. To minimize the bias of a training effect, in a cross-over like design half of the POT patients (*n* = 4) received M1-stimulation at day 1 and dMFC-stimulation at day 30, while the other half received M1- and dMFC-stimulation vice versa (i.e., M1-stimulation at day 30 and dMFC-stimulation at day 1)

Stimulation of either the left M1 area or dMFC was conducted on two separate sessions with an interval of at least one month in a cross-over design, i.e., half of the patient group received rTMS of M1 at the first session and rTMS of dMFC at the second session, the other half vice versa (for illustration see Fig. 1). All previous medications were continued during the study period.

Each patient underwent surface EMG from both lower legs and posturography (EO, EC, ECF) at each session before rTMS (pre-stim), and 20 min after rTMS (post-stim). Individual patients rated changes in their subjective feeling of postural stability during M1-rTMS and dMFC-rTMS by a scoring system from −3 to + 3 (−3: marked worsening; −2: moderate worsening; −1: slight worsening; 0: no change; + 1: slight improvement; + 2: moderate improvement; + 3: marked improvement).

### Data analysis

The obtained surface EMG recordings were analyzed for tremor frequency (Hz) and cumulative tremor intensity (micro-Volt*second, uv.s) calculated as area under the curve in each individual patient pre-stim and post-stim during EOF at both sessions (M1-rTMS and dMFC-rTMS). The posturography measurements (EOF condition) were analyzed for overall sway path (m/min) in *x* and *y* directions at session 1 and 2 (pre- and post-stim).

### Statistical analysis

Data are reported as mean ± standard deviation (SD). Shapiro–Wilk test indicated normal distribution of data. A mixed-design two-way repeated measurement analysis of variance (rmANOVA) was conducted to determine the effect of rTMS stimulation site to changes (pre- vs. post-stim) in tremor intensity, frequency and postural stability/sway path. The presence of a significant pre-vs. post-stim main effect was further evaluated using paired sample one-tailed *t*-tests for each stimulation site (M1 and dMFC).

Wilcoxon signed-rank test was applied to evaluate relationships between subjective patient ratings and objective changes in sway path (pre- vs. post-stim) for each stimulation site. Results were considered significant for *p* < *0.05*. Data processing and statistical analysis was performed using MATLAB® 2012a (Mathworks, Natick, MA, USA) software.

### Ethical standard

All subjects gave their informed, written consent to participate in the study. The study protocol was approved by the local ethics committee of the Ludwig-Maximilians-University of Munich and the study was in accordance with the Declaration of Helsinki.

## Results

Mean duration of POT in the study cohort was 13.5 ± 5.4 years, the median BDI-II score was 18 (range 7–29), and the median DHI-score was 61 (range 35–75). Seven of the eight POT patients were under treatment with either gabapentin (*n* = 5), clonazepam (*n* = 1) or propranolol (*n* = 1) (Table 1).

### M1-stimulation effects on tremor frequency, tremor intensity, and sway path

**Fig. 2:**
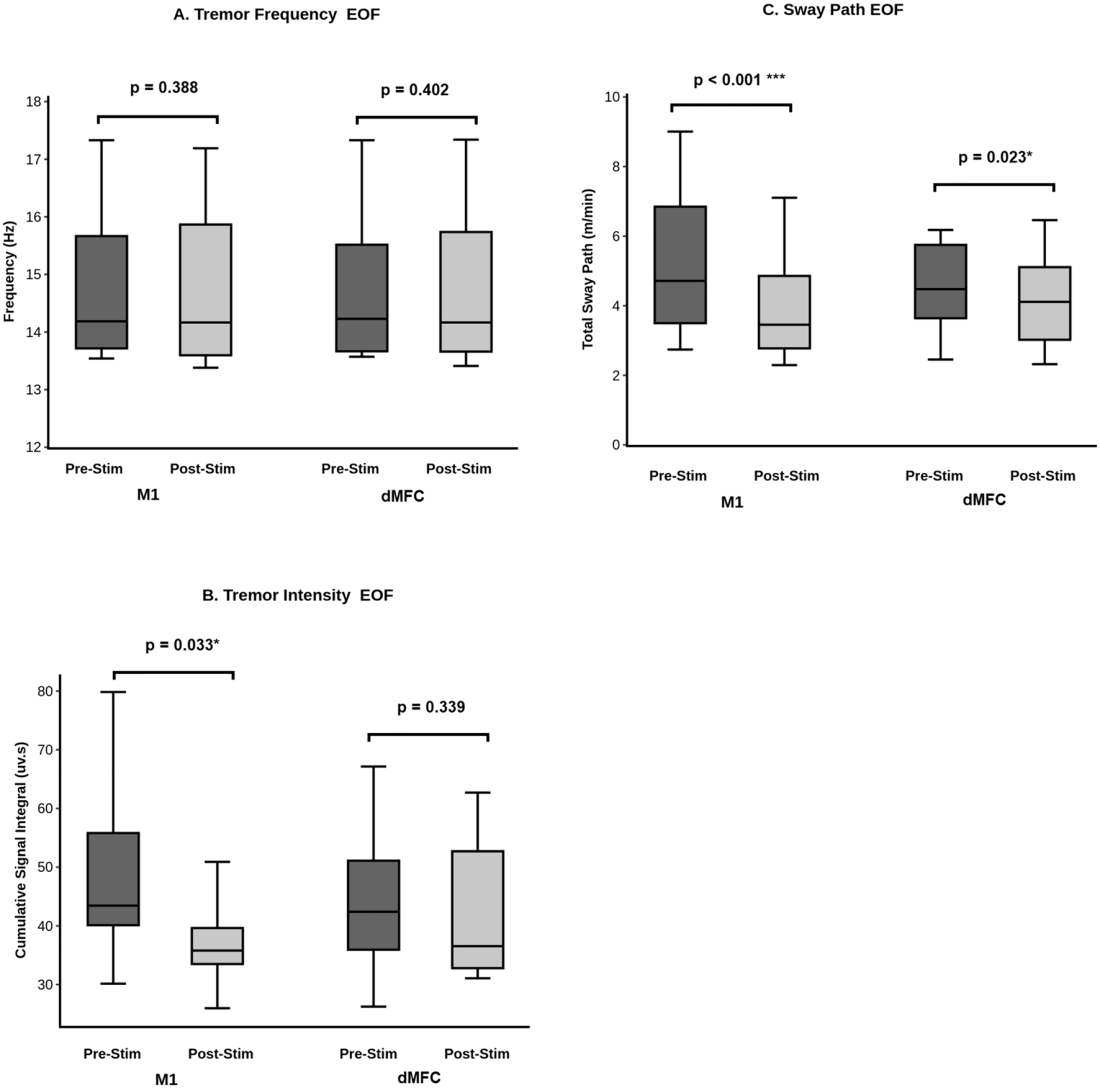
Statistical comparison of tremor frequency, intensity and sway path before and after M1- and dMFC-stimulation for the whole POT group. **A** Tremor frequency (Hz), **B** tremor intensity (uv.s), and **C** sway path (m/min) while standing on foam rubber with eyes closed (EOF), respectively depicted as whisker blots (mean, 25%/75% interquartils and standard deviation) before and after M1- and dMFC-stimulation

**Fig. 3:**
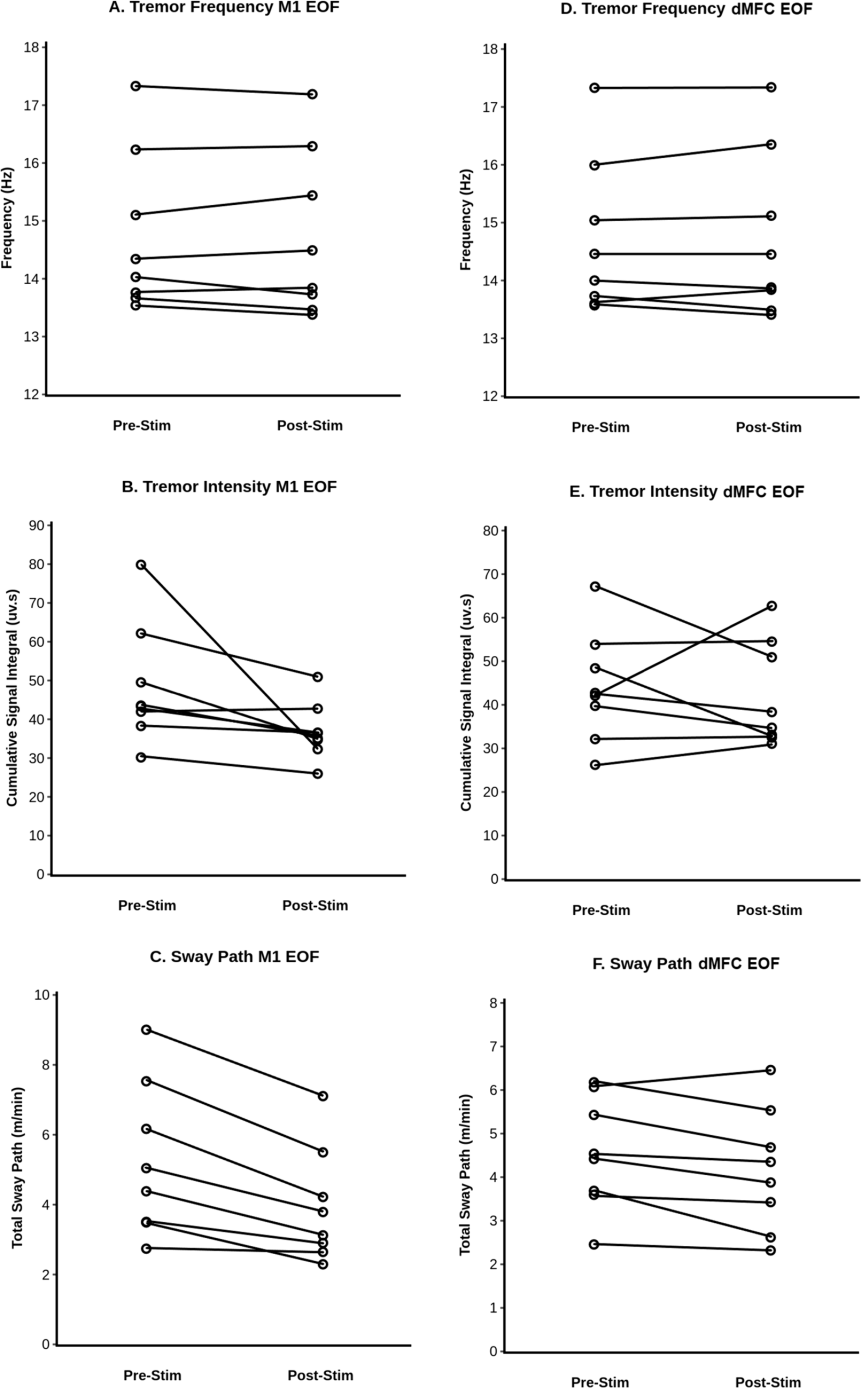
Illustration of the changes of tremor frequency, intensity and sway path in each individual POT patient after M1- and dMFC-stimulation.** A** Tremor frequency (Hz), **B** tremor intensity (uv.s), and **C** sway path (m/min) while patients were standing on foam rubber with eyes open (EOF) before and after M1-stimulation. **D** Tremor frequency, **E** tremor intensity, and **F** sway path (m/min) with EOF standing condition before and after dMFC-stimulation

Theta burst rTMS of the left M1 leg area did not change tremor frequency of POT at a group level (pre-stim: 14.75 ± 1.38 Hz vs. post-stim: 14.73 ± 1.43 Hz; *T* = *0.296, p* = *0.388*) (Table 2, Fig. 2A) nor in individual patients (Fig. 3A). There was a decrease in tremor intensity, i.e. the cumulative area under the curve of all the registered tremor bursts (pre-stim: 48.60 ± 15.60 vs. post-stim: 36.84 ± 7.37 uv.s; *T* = *2.175, p* = *0.033*) for M1-stimulation across the entire patient group (Fig. 2B). Accordingly, seven of eight patients showed a reduced tremor intensity comparing pre-/post-stim (Fig. 3B). The sway path on the posturography platform was significantly reduced after M1-stimulation on a group-level (pre-stim: 5.23 ± 2.18 vs. post-stim: 3.95 ± 1.63 m/min; *T* = *4.11, p* = *0.0005*) (Fig. 2C). Individually, seven of eight patients exhibited a significant decrease in sway path after M1-stimulation with only one patient showing neither improvement nor worsening (Fig. 3C).

**Table 2:** Tremor frequency, intensity, sway path pre- vs. post-stim for M1-/dMFC-rTMS

Stimulation site and applied statistics	Tremor frequency (Hz)	Tremor intensity (uv.s)	Sway path (m/min)
M1			
Pre-stim	14.75 ± 1.38	48.60 ± 15.60	5.23 ± 2.18
Post-stim	14.73 ± 1.43	36.84 ± 7.37	3.95 ± 1.63
dMFC			
Pre-stim	14.72 ± 1.35	44.03 ± 12.71	4.55 ± 1.30
Post-stim	14.73 ± 1.44	42.24 ± 12.07	4.16 ± 1.41
rmANOVA; Stimulation site x Time (*F, p*)	0.154, 0.700	2.137, 0.166	9.606, 0.008**
M1 paired *t*-test (*T, p*)	0.296, 0.388	2.175, 0.033	5.321, 0.0005**
dMFC paired *t*-test (*T, p*)	0.259, 0.401	0.433, 0.339	2.420, 0.023*

*M1* primary motor cortex (leg area), *dMFC* dorsal medial frontal cortex, *p<0.05, **p<0.01

All but one patient subjectively perceived an improvement of postural stability with M1-stimulation (slight improvement, i.e., rating + 1 in two patients, moderate improvement, i.e., rating + 2 in five patients) (supplementary data). Subjective patient rating of change in postural stability correlated with decrease in sway path after M1-rTMS (*Z* = −*2.46, p* = *0.014*).

### dMFC-stimulation effects on tremor frequency, tremor intensity, and sway path

dMFC-rTMS did not result in a change of tremor frequency (pre-stim: 14.72 ± 1.35 vs. post-stim: 14.73 ± 1.44 Hz; *T* = *0.259, p* = *0.401*) nor in tremor intensity in the entire POT group (pre-stim: 44.03 ± 12.71 vs. post-stim: 42.24 ± 12.07 uv.s; *T* = *0.433, p* = *0.339*) (Table 2, Fig. 2A). On an individual patient level, tremor frequency remained stable (Fig. 3D). Tremor intensity decreased in four patients, remained unchanged in two patients, and increased in two patients post-stim vs. pre-stim (Fig. 3E). In the entire group, sway path decreased significantly due to dMFC-rTMS (pre-stim: 4.55 ± 1.30 vs. post-stim: 4.16 ± 1.41 m/min; *T* = *2.42; p* = *0.023*). Four patients showed a decrease in sway path, while three patients had no effect and one patient had a higher sway path after dMFC-stimulation (Fig. 3F). Subjective rating of postural stability revealed an improvement in six patients (slight improvement in five patients, moderate improvement in one patient) upon dMFC-stimulation (supplementary data), which correlated with decrease of sway path (*Z* = −2.330*, p* = *0.02*).

## Discussion

The present study examined the effects of theta burst rTMS in the left primary motor cortex and dorsal medial frontal cortex on tremor characteristics, sway path, and subjective feeling of postural stability in a well characterized cohort of patients with primary orthostatic tremor. The key result was that tremor intensity improved with M1- but not dMFC-rTMS. M1-stimulation and to a lesser extent dMFC-stimulation decreased sway path signficantly. Consistently with this, the patients reported subjective improvement of stance stability. The underlying mechanisms and practical relevance of these findings will be discussed in the following sections.

### Influence of theta burst M1-/dMFC-rTMS on tremor characteristics in POT

In this study, rTMS of M1 and dMFC had differential effects on POT characteristics. Tremor frequency remained unchanged for both conditions, while intensity decreased on the group level after M1-rTMS only (corresponding to six of eight individual patients). Generally, it is conceivable that M1-stimulation had more pronounced effects on tremor compared to dMFC-stimulation, as it more directly interferred with the known cortical represenations of the tremor network underlying POT [18]. Thereby, presumably inhibitory M1-rTMS may attenuate the oscillatory activity in the tremor network, but may not change its inherent frequency. This view is in accordance with previous reports in single patients with POT that described no effect of suprathreshold TMS of the primary motor cortex on tremor frequency, but an immediate diminution of tremor intensity [12]. Similarly, DBS in the ventral intermediate thalamus (VIM) or zona incerta (ZI) decreased tremor intensity significantly in 20 patients with POT, despite having no impact on tremor frequency [14–17, 24–27]. Compared to the current study, suprathreshold TMS and VIM-/ZI-DBS effects on tremor intensity in the aforementioned studies seemed to be stronger. For TMS, this may be explained by differences in stimulation conditions and timescales of protocols. While we applied subthresthold rTMS with a stimulus intensity of 80% of the RMT for M1, in the previous studies the stimulus intensities were respectively 10% and 20% above the RMT. In addition, rTMS in the current study was administered only unilaterally for methodological reasons, while the tremor network in POT is represented bihemispherically. Furthermore, we analyzed treatment effects not directly, but 20 min after theta burst rTMS application. It therefore can be presumed that the effects of theta burst rTMS in our study may have been underestimated and tremor during or immediately after M1-rTMS was partially or completely suppressed. As far as VIM-/ZI-DBS is concerned, we speculate that a persistent, invasive and direct modulation of a hub in the ponto-cerebello-thalamo-cortical tremor network in POT [18, 19, 28] unfolds more pronounced effects on the tremor intensity than one train of theta burst rTMS for 40 s in our study.

### Putative mechanism of theta burst M1/dMFC-rTMS on postural stability in POT

Subjectively perceived and objectively measured postural stability significantly improved following M1-rTMS. dMFC-stimulation also resulted in a decrease of the overall sway path and increase in subjective postural stability, but to a lesser extent than M1-stimulation.

The putative mechanism behind the positive effect of M1-stimulation on balance control might be desynchronization of the entire ponto-cerebello-thalamo-cortical oscillatory network underlying POT by inhibition of activity in its motor cortical core hub [18, 19, 28] [18, 25, 29, 30]. Positive effects of transcranial neuromodulation have also been described for other common tremor disorders, such as essential tremor (ET) and Parkinsonian tremor (PT), which share an oscillating loop involving sensorimotor cortical areas [31–35]. Since M1-rTMS in POT decreased the tremor intensity, the pronounced improvement of postural stability could be seen as an immediate consequence of tremor attenuation. However, in POT the correlation of tremor intensity and postural instability is not that unequivocal. Previous studies have either reported a disproportional increase of postural unsteadiness when compared to tremor severity [20, 36], or a marked improvement of postural control despite no relevant change in tremor intensity [37]. These observations point towards somewhat distinct albeit partially overlapping circuits for tremor generation and postural control in POT. On a cortical level, deactivation of mesio- and prefrontal areas correlates more intensely than primary motor areas with the extent of postural sway in POT [18]. In line with this, functional imaging studies in healthy subjects have indicated activations of both primary motor and premotor/supplementary motor cortical regions during stance [38–42]. Interestingly, more complex balance tasks seem to trigger the activation of premotor and supplementary motor areas more extensively than the primary motor area [43]. In patients with stroke lesions, involvement of the dMFC is critically relevant for postural balance control and anticipatory postural adjustments [39, 44, 45].

This functional topography could explain why dMFC-rTMS resulted in an obvious dissociation between a significant decrease of sway path and perceived postural instability on the one side and an unaffected tremor intensity on the other. The dMFC seems not directly involved in the oscillating tremor network of POT and ET, but rather a modulating brain region for the cortico-thalamo-cerebellar core network [18, 31]. dMFC shows significant structural and functional changes in POT [18–20] with an increase in grey matter volume and functional coupling to cerebellar brain regions. Theta burst rTMS of cerebellar areas (particularly lobule VI) led to a decoupling of cerebellar-dMFC intrinsic activities as a possible underlying correlate of a decrease in POT severity [19]. Based on these observations, there is enough pathophysiological evidence that the dMFC might be a possible target for non-invasive neuromodulation in POT as well as postural balance control deficits generally.

In conclusion, the present study gives evidence that non-invasive transcranial stimulation of the leg area of the primary motor cortex might improve both tremor intensity and postural control in POT and could be a potential neuromodulatory add-on therapy for this rather hard to treat rare disorder. Effects of M1-rTMS on postural stability surpassed the benefit of dMFC-rTMS. Targeting postural instability addresses the need that affected patients often perceive imbalance as the most disabling chief complaint. However, additional studies with further control conditions, a longer treatment duration, or a bilateral stimulation protocol are urgently needed to make a final statement on rTMS effectiveness, especially compared to previously described methods such as peripheral somatosensory stimulation, spinal cord stimulation, or DBS [46–48].

### Supplementary Information

Below is the link to the electronic supplementary material.

Supplementary file1 (DOCX 13 KB)

## Data Availability

The datasets used and analysed during the current study are available from the corresponding author on reasonable request.

## Abbreviations

BDI: Beck depression inventory
DBS: Deep brain stimulation
DHI: Dizziness Handicap Inventory
dMFC: Dorsal medial frontal cortex
EC: Eyes closed
EEG: Electroencephalography
EMG: Electromyography
EO: Eyes open
EOF: Eyes open foam rubber
ET: Essential tremor
M1: Primary motor cortex
OT: Orthostatic tremor
POT: Primary orthostatic tremor
PT: Parkinsonian tremor
RMS: Resting motor threshold
rTMS: Repetitive transcranial magnetic stimulation
VIM: Ventral intermediate (thalamus)
ZI: Zona incerta

## Code availability

The underlying code for this study is not publicly available but may be made available to qualified researchers on reasonable request from the corresponding author.
