# Supplementary material for: TMS of the left primary motor cortex improves tremor intensity and postural control in primary orthostatic tremor
Source: J Neurol. 2024 Apr 16;271(6):2938–47. doi: 10.1007/s00415-024-12376-3 (PMC11136716; doi:10.1007/s00415-024-12376-3)
Supplement: Supplementary file 1 — Supplementary file1 (DOCX 13 KB) [file 415_2024_12376_MOESM1_ESM.docx]

**Supplementary data**

**Supplementary table** Subjective rating of postural stability

| **ID** | **Sex** | **Age** | **POT duration (years)** | **Subjective rating after M1-rTMS** | **Subjective rating after dMFC-rTMS** |
| --- | --- | --- | --- | --- | --- |
| 1 | f | 57 | 8 | +2 | +1 |
| 2 | f | 71 | 23 | +1 | +1 |
| 3 | m | 77 | 14 | 0 | 0 |
| 4 | f | 58 | 11 | +1 | 0 |
| 5 | f | 68 | 18 | +2 | +1 |
| 6 | m | 76 | 13 | +2 | +1 |
| 7 | m | 78 | 15 | +2 | +1 |
| 8 | m | 59 | 6 | +2 | +2 |

Scoring system from -3 to +3 (-3: marked worsening; -2: moderate worsening; -1: slight worsening; 0: no change; +1: slight improvement; +2: moderate improvement; +3: marked improvement).
